# Clinical characteristics and exploratory serum biomarker findings in vestibular migraine: a cross-sectional case–control study

**DOI:** 10.3389/fneur.2026.1929972

**Published:** 2026-08-26

**Authors:** Bingyang Liu, Daopei Zhang, Lili Zhang, Yonghui Zhang, Xiaofang Guan

**Affiliations:** 1 Department of Encephalopathy, The First Affiliated Hospital of Henan University of Chinese Medicine, Zhengzhou, Henan, China; 2 The First Clinical Medical College, Henan University of Chinese Medicine, Zhengzhou, Henan, China; 3 The First Affiliated Hospital of Tianjin University of Chinese Medicine, Tianjin, China; 4 Henan Vertigo Diagnosis and Treatment Center, Zhengzhou, Henan, China; 5 National Clinical Research Center for Chinese Medicine, Tianjin, China

**Keywords:** vestibular migraine, clinical characteristics, serum biomarkers, calcitonin gene-related peptide, growth differentiation factor-15

## Abstract

**Objective:**

Vestibular migraine (VM) is a common disorder in vertigo clinics, but its diagnosis remains challenging because of symptom overlap with other conditions and the lack of widely accepted objective biomarkers. This exploratory cross-sectional case–control study aimed to characterize the clinical features of VM, examine clinical and serum factors associated with VM case status, and assess the apparent ability of serum biomarkers to distinguish VM cases from healthy controls.

**Methods:**

Between May and December 2025, 145 patients with VM and 50 healthy controls were enrolled at the First Affiliated Hospital of Henan University of Chinese Medicine. Demographic characteristics, clinical symptoms, triggering factors, family history, previous diagnoses, and self-reported medical costs were collected. Dizziness, headache impact, anxiety and depressive symptoms, and cognitive screening performance were assessed using the DHI, H-VAS, HIT-6, HAMA-14, HAMD-17, and MoCA-BJ. Serum CGRP, GDF-15, FGF-21, and IL-17 were measured in a biomarker subset of 60 VM cases and 20 healthy controls. Univariable and exploratory multivariable logistic regression analyses examined associations with VM case status, and ROC analysis assessed discrimination between VM cases and healthy controls within the biomarker development sample.

**Results:**

The male-to-female ratio among VM patients was 1:6.6. Emotional fluctuations, fatigue, and sleep deprivation were frequently reported triggers. Anxiety or depression (40.0%) and cervical spondylosis (27.6%) were the most commonly reported previous diagnoses. In separate exploratory multivariable models, female sex, higher BMI, and a positive family history remained associated with VM case status in the full clinical cohort, whereas hypertension was not statistically significant after adjustment. In the biomarker subset, higher serum CGRP and GDF-15 levels remained associated with VM case status, whereas FGF-21 was not significantly associated; the apparent AUCs were 0.794 for CGRP, 0.898 for GDF-15, and 0.927 for their combination. The combined AUC was higher than that for CGRP alone (DeLong *p* = 0.006) but not significantly higher than that for GDF-15 alone (*p* = 0.227).

**Conclusion:**

VM in this single-center cohort was characterized by female predominance, recurrent symptoms, substantial symptom burden, and familial aggregation. Female sex, higher BMI, and a positive family history were clinical factors associated with VM case status after adjustment. In the biomarker subset, higher serum CGRP and GDF-15 levels were associated with VM case status, and the two biomarkers showed exploratory ability to discriminate VM cases from healthy controls.

## 1 Introduction

Vestibular migraine (VM) is a common condition characterized by the coexistence of migraine features and vestibular dysfunction. Its core manifestations include episodic dizziness or vertigo, often accompanied by nausea, vomiting, headache, and other migraine-related symptoms; familial aggregation has also been reported (1). VM can substantially impair daily activities, work, and study and is frequently accompanied by anxiety, depressive symptoms, and sleep disturbance, thereby reducing quality of life (2). Epidemiological studies have reported VM in 4.2–29.3% of patients presenting with dizziness or vertigo and in 10.3–21.0% of patients with migraine (3). A population-based survey in the United States using ICHD-3 criteria reported an adult prevalence of approximately 2.7% (4, 5). VM is therefore recognized as one of the most common disorders encountered in vertigo clinics.

Because VM symptoms overlap with those of benign paroxysmal positional vertigo (BPPV), Ménière’s disease, posterior circulation disorders, persistent postural-perceptual dizziness (PPPD), and psychiatric conditions, diagnosis may be delayed or incorrect (6, 7). The pathophysiology remains incompletely understood. Proposed mechanisms include cortical spreading depression, trigeminovascular activation, altered central sensory integration, ion-channel dysfunction, and neuropeptide dysregulation (8, 9). Calcitonin gene-related peptide (CGRP) is involved in migraine-related trigeminovascular signaling, whereas growth differentiation factor-15 (GDF-15), fibroblast growth factor 21 (FGF-21), and interleukin-17 (IL-17) are broader stress, metabolic, or inflammatory biomarkers rather than neurotransmitters (10, 11).

Evidence regarding serum biomarkers in VM remains preliminary, and few studies have evaluated these markers in well-characterized clinical cohorts. The present exploratory study therefore described the demographic and clinical phenotype of VM, examined factors associated with VM case status, and assessed serum CGRP, GDF-15, FGF-21, and IL-17 in VM cases and healthy controls. Because the control group did not include patients with other vestibular disorders, the biomarker analyses were intended to estimate case–control discrimination rather than clinical differential-diagnostic performance.

## 2 Materials and methods

### 2.1 Study subjects and grouping

This was a single-center, cross-sectional case–control study. Patients with VM who met the inclusion criteria were consecutively recruited from the outpatient clinic of the Department of Encephalopathy, the First Affiliated Hospital of Henan University of Chinese Medicine, between May and December 2025. Healthy controls undergoing health examinations were recruited during the same period and were frequency-matched by age. The study was approved by the hospital Ethics Committee (No. 2025HL-547-01), and all participants provided written informed consent.

### 2.2 Diagnostic, inclusion and exclusion criteria

VM was diagnosed according to the 2022 Bárány Society/International Headache Society criteria and the International Classification of Headache Disorders, third edition (ICHD-3) (12, 13). The enrolled cohort included patients meeting criteria for definite or probable VM, and all diagnoses were independently confirmed by two senior neurologists. Participants were aged 18 years or older. Exclusion criteria included major organ failure, malignant tumors, autoimmune disease, cerebrovascular disease, neurodegenerative disorders, other structural brain disorders, alternative causes of vestibular symptoms, and incomplete clinical data.

### 2.3 Definition and assessment of positive family history

Positive family history was defined as participant-reported headache/migraine and/or recurrent dizziness/vertigo among first- or second-degree relatives. Family history was coded as 1 = positive and 2 = negative and analyzed as a single combined variable rather than as separate headache and dizziness variables. Where sufficient information was available, symptoms attributable to clear secondary causes were excluded. This classification was used to describe familial aggregation and was not considered direct evidence of genetic susceptibility.

### 2.4 Sample grouping process

**Figure 1:**
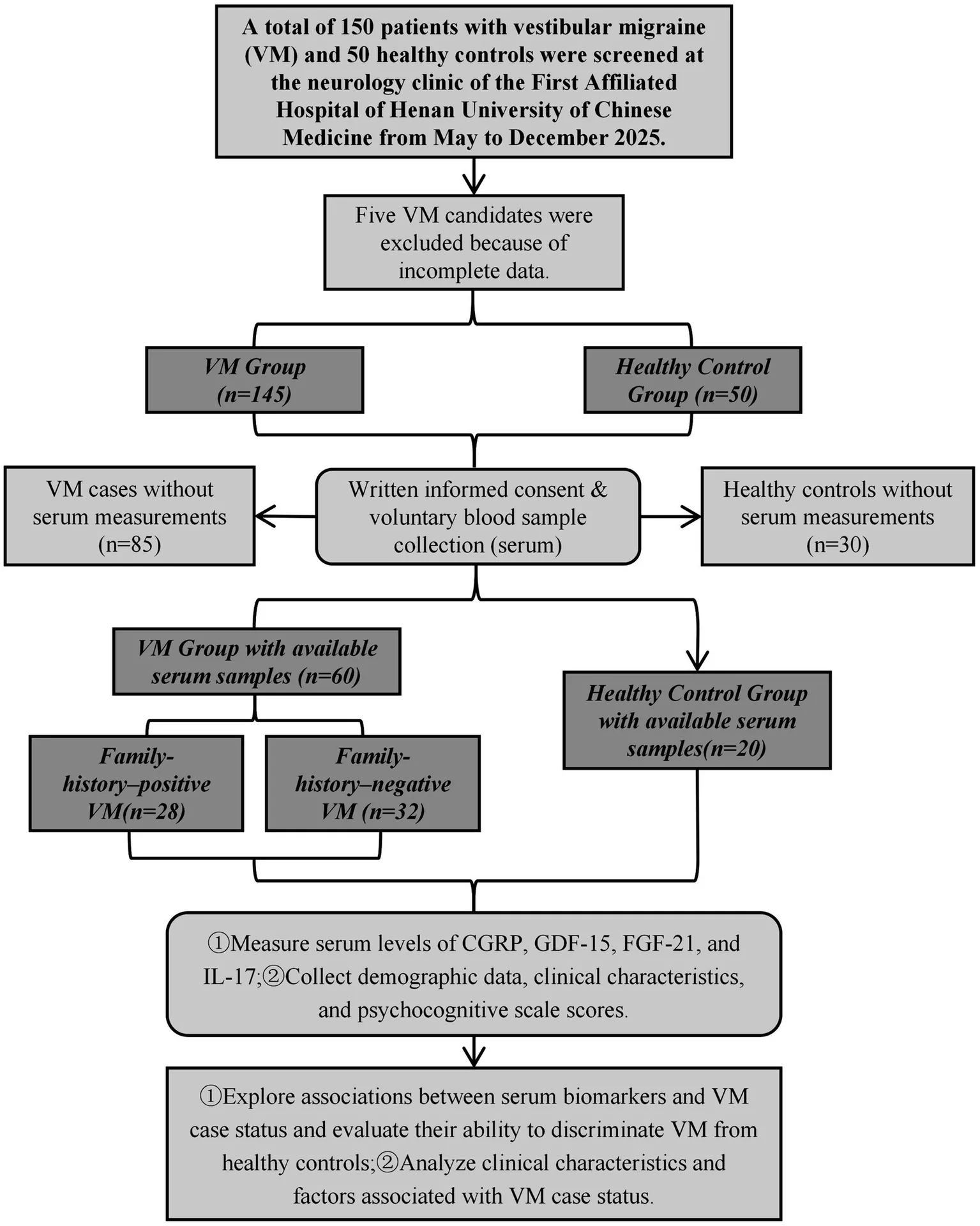
Participant flow diagram. CGRP, Calcitonin gene-related peptide; GDF-15, Growth differentiation factor-15; FGF-21, Fibroblast growth factor 21; IL-17, Interleukin-17.

The main clinical analysis included 145 patients with VM and 50 healthy controls. Serum biomarker measurements were available for 60 VM cases and 20 healthy controls, whereas 85 VM cases and 30 healthy controls had no available serum biomarker measurements. To assess potential selection differences related to optional serum sampling, participants with and without available serum measurements were compared separately among VM cases and healthy controls. The detailed study grouping process is illustrated in Figure 1.

### 2.5 Data collection and assessment

#### 2.5.1 General data questionnaire

A self-designed standardized questionnaire was used to collect demographic data (sex, age, height, weight, and educational level), disease duration, core and accompanying symptoms, triggering or aggravating factors, medical history, family history, relevant laboratory and imaging findings, previous diagnoses, and medical burden. Vascular factors included hypertension, diabetes mellitus, coronary heart disease, atrial fibrillation, hyperlipidemia, hyperuricemia, and previous stroke. Body mass index (BMI) was calculated from measured height and weight. Previous alternative diagnoses were defined as diagnoses documented in available medical records or reported by participants before the final VM diagnosis. Medical burden was defined as self-reported direct healthcare expenditure related to dizziness, vertigo, or headache before enrollment; the observation period and individual cost components were not standardized.

#### 2.5.2 Related scale assessment

Dizziness Handicap Inventory (DHI) (14): Used to evaluate the severity of dizziness and its impact on daily life. The scale includes 3 subscales: physical (P, 7 items), emotional (E, 9 items) and functional (F, 9 items). It is graded as follows: 0–30 points, mild impairment; 31–60 points, moderate impairment; 61–100 points, severe impairment.

Headache-Visual Analogue Scale (H-VAS) (15): Widely used for pain assessment. Headache severity was rated numerically from 0 to 10 (0 = no pain, 10 = severe headache) according to the Guidelines for the Diagnosis and Treatment of Migraine.

Headache Impact Test-6 (HIT-6) (16): Used to evaluate the impact of headache on quality of life, covering 6 dimensions: pain, social function, social role, vitality, cognitive function, and psychological disorder. The HIT-6 total score ranges from 36 to 78 and was interpreted as follows: ≤49, no or mild impact; 50–55, moderate impact; 56–59, significant impact; and ≥60, severe impact.

Hamilton Anxiety Rating Scale (HAMA-14) (17, 18): The Chinese 14-item HAMA was used to assess anxiety symptom severity. Each item was rated from 0 to 4, yielding a total score of 0–56. Scores were interpreted as follows: <7, no clinically relevant anxiety symptoms; 7–13, possible anxiety symptoms; 14–20, definite anxiety symptoms; 21–28, marked anxiety symptoms; and ≥29, severe anxiety symptoms. The scale was not used alone to establish a psychiatric diagnosis.

Hamilton Depression Rating Scale (HAMD-17) (19): The Chinese 17-item HAMD was used to assess depressive symptom severity. Scores were interpreted as follows: <7, no clinically relevant depressive symptoms; 7–17, possible depressive symptoms; 18–24, definite depressive symptoms; and >24, severe depressive symptoms. The scale was not used alone to establish a psychiatric diagnosis.

Montreal Cognitive Assessment, Beijing version (MoCA-BJ) (20): The MoCA-BJ was used to screen global cognitive function. One additional point was added for participants with ≤12 years of formal education, with the total score capped at 30. A score below 26 was regarded as a positive cognitive screening result requiring further assessment rather than as a stand-alone diagnosis of cognitive impairment.

#### 2.5.3 Serum sampling and biomarker measurement

After informed consent, venous blood was collected during the interictal period, operationally defined as the absence of an ongoing vestibular or migraine attack at the time of blood collection. The exact interval from the most recent attack was not systematically recorded. Whole-blood samples were allowed to stand at room temperature for 2 h or at 4 °C overnight and were then centrifuged at 2–8 °C and 3,000 rpm for 15 min. Serum was separated, aliquoted, and stored at −80 °C; repeated freeze–thaw cycles were avoided, and thawed samples were centrifuged again before testing. Serum biomarkers were measured using sandwich ELISA kits: human CGRP1 (Elabscience, catalogue No. E-EL-H0619), human IL-17A (E-EL-H5812), human GDF-15 (E-EL-H0080), and human FGF-21 (E-EL-H0074). Assays were performed according to the manufacturers’ instructions. Standard, blank, and sample wells were established; after sequential incubation with biotinylated detection antibody and horseradish-peroxidase conjugate, the plates were washed, developed with TMB substrate, stopped, and read at 450 nm. Standard curves were generated using Origin 2021, and sample concentrations were calculated from the fitted equations. Laboratory personnel performing the assays were blinded to participants’ clinical group allocation.

#### 2.5.4 Auxiliary examinations

All patients with VM underwent positional testing, video head impulse testing (v-HIT), bithermal caloric testing, vestibular-evoked myogenic potential testing, hearing assessment, and cranial imaging as clinically indicated. The v-HIT was used primarily to assess high-frequency semicircular-canal vestibulo-ocular reflex function, whereas caloric testing was performed to evaluate low-frequency horizontal semicircular-canal function. The complementary use of v-HIT and caloric testing enabled a more comprehensive assessment of semicircular-canal function. Abnormal findings were reviewed in conjunction with the clinical history, audiological findings, and imaging results to identify or exclude other vestibular disorders.

### 2.6 Study methods

Trained researchers provided standardized instructions for completion of questionnaires and scales, which were collected on site and checked for completeness. The main clinical analyses used 145 VM cases and 50 healthy controls, whereas biomarker analyses were restricted to the 60 VM cases and 20 healthy controls with available serum measurements. Clinical symptoms, triggering factors, previous diagnoses, and medical burden were analyzed among patients with VM.

### 2.7 Statistical analysis

Statistical analyses were performed using SPSS version 26.0 (IBM Corp., Armonk, NY, USA) and R version 4.5.0 (R Foundation for Statistical Computing, Vienna, Austria). DeLong comparisons, bootstrap internal validation with 1,000 resamples, and calibration analyses were conducted in R. Categorical variables are presented as *n* (%) and were compared using the chi-square test or Fisher’s exact test, as appropriate. Continuous variables were assessed for normality using the Shapiro–Wilk test. Normally distributed data are presented as mean ± standard deviation and were compared using the independent-samples t-test. Non-normally distributed data are presented as median with interquartile range (IQR) and were compared using the Mann–Whitney U test or Kruskal-Wallis *H* test, as appropriate. Significant three-group comparisons were followed by Dunn-Bonferroni pairwise tests, with *p* < 0.0167 considered significant.

To assess potential selection differences related to optional serum sampling, participants with and without available serum biomarker measurements were compared separately among VM cases and healthy controls. Continuous variables were compared using the independent-samples t-test or the Mann–Whitney U test according to their distributions, whereas categorical variables were compared using Pearson’s chi-square test or Fisher’s exact test, as appropriate. Standardized mean differences (SMDs) were also calculated to quantify the magnitude of subgroup imbalance. An absolute SMD ≥ 0.10 was considered indicative of a potentially meaningful subgroup imbalance.

Univariable comparisons were performed to identify variables associated with VM case status. Variables with *p* < 0.05 in the univariable analyses were considered for model-specific multivariable analyses. Two separate exploratory multivariable logistic regression models were fitted because clinical variables were available in the full cohort, whereas serum biomarkers were available only in a subset. For logistic regression analyses, VM case status was coded as 1 and healthy-control status as 0. Female sex, hypertension, and positive family history were coded as 1, whereas male sex, absence of hypertension, and negative family history were coded as 0 and served as the reference categories. The clinical model included age, female sex, BMI, hypertension, and positive family history in the full cohort of 145 VM cases and 50 healthy controls. Although controls were frequency-matched by age, age was additionally retained as a covariate to account for residual age variation. The biomarker model included CGRP, FGF-21, and GDF-15 in the subset of 60 VM cases and 20 healthy controls. All variables within each model were entered simultaneously. ROC analysis assessed discrimination between VM cases and healthy controls. AUC, 95% *CI*, sensitivity, specificity, and optimal cut-off values based on the maximum Youden index were calculated. AUCs were compared using the DeLong test. The combined CGRP plus GDF-15 model was internally evaluated using 1,000 bootstrap resamples, with optimism-corrected AUC, calibration intercept, and calibration slope reported. All tests were two-sided, and *p* < 0.05 was considered statistically significant unless otherwise specified.

## 3 Results

### 3.1 General characteristics

Among the 145 patients with VM, 19 (13.1%) were male and 126 (86.9%) were female, yielding a male-to-female ratio of approximately 1:6.6. Patient age ranged from 18 to 75 years, with a mean of 48.55 ± 12.77 years. The age distribution comprised 38 young adults (26.2%, 18–44 years), 89 middle-aged individuals (61.4%, 45–59 years), and 18 elderly patients (12.4%, ≥60 years). Mean BMI was 24.66 ± 3.15 kg/m^2^, approaching the threshold for overweight. In terms of educational attainment, the largest subgroup had completed junior high school (60 cases, 41.4%), followed by senior high school (38 cases, 26.2%), undergraduate studies (22 cases, 15.2%), primary school (23 cases, 15.9%), and postgraduate studies (2 cases, 1.4%).

### 3.2 Clinical features

#### 3.2.1 Disease course and core symptoms

The disease duration in patients with VM was most often over 10 years (42 cases, 28.9%), followed by less than 1 year (31 cases, 21.4%), 1–3 years (25 cases, 17.2%), 5–10 years (24 cases, 16.6%), and 3–5 years (23 cases, 15.9%). Nearly 80% of patients had a disease duration exceeding 1 year, indicating that VM follows a protracted course with a propensity for long-term recurrent episodes (Table 1).

**Table 1:** Clinical characteristics of 145 patients with VM.

Item	Number of cases (%)
Disease duration distribution	
<1 year	31 (21.4)
1–3 years	25 (17.2)
3–5 years	23 (15.9)
5–10 years	24 (16.6)
>10 years	42 (28.9)
Core symptoms	
Alternating episodes of vertigo/dizziness and headache	101 (69.7)
Vertigo/dizziness combined with headache	36 (24.9)
Isolated vertigo/dizziness	8 (5.5)
Duration of vertigo/dizziness episodes	
A few seconds	2 (1.4)
A few seconds to a few minutes	37 (25.5)
A few minutes to a few hours	58 (40.0)
A few hours to 1 day	33 (22.8)
Several days	15 (10.3)
Duration of headache episodes	
A few seconds	1 (0.7)
A few seconds to a few minutes	8 (5.5)
A few minutes to a few hours	48 (33.1)
A few hours to 1 day	73 (50.3)
Several days	15 (10.3)
Frequency of vertigo/dizziness episodes	
<5 episodes	28 (19.3)
≥5 episodes	63 (43.4)
Frequent episodes	54 (37.2)
Frequency of headache episodes	
<5 episodes	22 (15.2)
≥5 episodes	67 (46.2)
Frequent episodes	56 (38.6)

Core symptoms most commonly consisted of alternating vertigo/dizziness and headache (101/145, 69.7%), followed by concurrent vertigo/dizziness and headache (36/145, 24.9%); 8 patients (5.5%) reported isolated vertigo/dizziness. Vertigo/dizziness episodes most often lasted from minutes to hours, and headache episodes most often lasted from hours to 1 day. Most participants reported at least five or frequent recurrent attacks (Table 1).

#### 3.2.2 Accompanying symptoms

All 145 VM patients presented with varying degrees of accompanying symptoms. Among the top seven symptoms, each had an incidence rate of at least 73.8%, with nausea and/or vomiting accounting for the highest proportion (123 cases, 84.8%), followed by fatigue (122 cases, 84.1%), irritability (120 cases, 82.8%), neck stiffness and discomfort (118 cases, 81.4%), and sadness or low mood (111 cases, 76.6%). Blurred vision and sleep disturbance were tied for the sixth most common symptom, each occurring in 107 cases (73.8%) (Table 2). Other common accompanying symptoms included chest tightness and palpitations, dry mouth, phonophobia, tinnitus and photophobia. Hearing loss and aural fullness were relatively less frequent. Among the 145 patients with VM, 26 (17.9%) experienced visual aura.

**Table 2:** Accompanying symptoms in patients with VM.

Accompanying symptom	Number of cases (%)
Nausea and/or vomiting	123 (84.8)
Fatigue	122 (84.1)
Irritability	120 (82.8)
Neck stiffness or discomfort	118 (81.4)
Low mood or sadness	111 (76.6)
Blurred vision	107 (73.8)
Sleep disturbance	107 (73.8)
Chest tightness and palpitations	97 (66.9)
Phonophobia	94 (64.8)
Tinnitus	84 (57.9)
Photophobia	82 (56.6)
Hearing loss	56 (38.6)
Ear fullness	55 (37.9)
Visual aura	26 (17.9)

#### 3.2.3 Predisposing or exacerbating factors

Among the 145 patients with VM, only one patient (0.7%) reported no identifiable triggering or aggravating factor, whereas triggers or exacerbating conditions were identified in 99.3% of patients. Psychosocial factors accounted for the largest proportion, with the top three triggers being emotional fluctuations (stress/anger/pressure, 130 cases, 89.6%), fatigue (128 cases, 88.3%), and sleep deprivation (120 cases, 82.7%). In terms of dietary factors, most patients were triggered primarily by spicy or greasy foods (50 cases, 34.5%), and some experienced worsening symptoms after consuming coffee or alcohol. Among environmental triggers, bright light or noise (105 cases, 72.4%) and exposure to cold and/or heat (98 cases, 67.6%) were the main precipitating factors, while a smaller subset (27 cases, 18.6%) experienced onset or exacerbation during seasonal changes. Regarding physical factors, respiratory or gastrointestinal infections (71 cases, 49.0%) were relatively common. Menstruation was reported as a trigger by 57 of 126 female participants (45.2%; 39.3% of the total VM cohort). The detailed classification and statistics are presented in Table 3.

**Table 3:** Precipitating and aggravating factors in patients with VM.

Reported triggering or aggravating factor	Number of cases (%)
Emotional fluctuations	130 (89.6)
Fatigue	128 (88.3)
Sleep deprivation	120 (82.7)
Dietary factors	
Alcohol	5 (3.4)
Caffeine	23 (15.9)
Spicy, irritating, or greasy food	50 (34.5)
Environmental factors	
Bright light or noise	105 (72.4)
Cold and/or heat exposure	98 (67.6)
Seasonal changes	27 (18.6)
Other factors	
Menstruation among women	57/126 (45.2)*
Respiratory or gastrointestinal infections	71 (49.0)

* This corresponds to 39.3% of the total VM cohort.

#### 3.2.4 Misdiagnosis and medical burden

Previous alternative diagnoses were common among patients with VM, and all 145 participants reported at least one previous alternative diagnosis before the diagnosis of VM. The most commonly reported previous alternative diagnosis was anxiety or depression (58 cases, 40.0%), followed by cervical spondylosis, BPPV, Ménière’s disease, and vestibular neuritis (VN) (Table 4). Regarding medical burden, the distribution of self-reported healthcare costs was as follows. The largest proportion of patients (55 cases, 37.9%) incurred costs between 2,000 and 5,000 RMB, followed by 10,000–50,000 RMB (36 cases, 24.8%) and 5,000–10,000 RMB (32 cases, 22.1%). Only 16 patients (11.0%) had medical costs ≤ 2,000 RMB, whereas 6 patients (4.1%) incurred costs ≥ 50,000 RMB. Cumulatively, 89.0% of patients had medical expenses exceeding 2,000 RMB, indicating that VM imposes a substantial economic burden on affected individuals.

**Table 4:** Previous alternative diagnoses and medical burden in patients with VM.

Item	Number of cases (%)
Previous alternative diagnoses	
Anxiety or depression	58 (40.0)
Cervical spondylosis	40 (27.6)
Benign paroxysmal positional vertigo	24 (16.5)
Ménière’s disease	10 (6.9)
Vestibular neuritis	5 (3.4)
Other previous diagnoses	8 (5.5)
Medical burden (cost, RMB)	
≤2,000	16 (11.0)
2,001-5,000	55 (37.9)
5,001-10,000	32 (22.1)
10,001-49,999	36 (24.8)
≥50,000	6 (4.1)

Each participant was classified according to the principal previous alternative diagnosis.

### 3.3 Comparison of scale scores between the two groups

All scale scores were non-normally distributed. Compared with healthy controls, VM cases had higher DHI, H-VAS, HIT-6, HAMA-14, and HAMD-17 scores and lower MoCA-BJ scores (all *p* < 0.001; Table 5). These findings indicate greater dizziness- and headache-related burden, more anxiety and depressive symptoms, and lower cognitive screening performance; they do not by themselves establish psychiatric or cognitive diagnoses.

**Table 5:** Comparison of scale scores between VM cases and healthy controls.

Item	VM group (*n* = 145)	Healthy controls (*n* = 50)	*Z*	*p* value
DHI	58 (52,60)	6 (4,6)	−10.563	< 0.001
H-VAS	6 (6,7)	1 (0,2)	−10.663	< 0.001
HIT-6	60 (55,62)	36.5 (36,38)	−10.567	< 0.001
HAMA-14	10 (7,12)	0 (0,2)	−10.411	< 0.001
HAMD-17	8 (5,12)	0 (0,2)	−10.022	< 0.001
MoCA-BJ	26 (24,27)	28 (27,29)	−6.713	< 0.001

Data are presented as median (IQR). HAMA-14, 14-item Hamilton Anxiety Rating Scale; HAMD-17, 17-item Hamilton Depression Rating Scale; MoCA-BJ, Beijing version of the Montreal Cognitive Assessment.

### 3.4 Comparison of serum biomarkers by family-history status and healthy-control status

Serum biomarker data were available for 60 patients with VM and 20 healthy controls. Using the combined family-history variable, patients with VM were categorized as family-history positive (n = 28) or family-history negative (*n* = 32).

Kruskal–Wallis tests showed significant differences in CGRP (*H* = 15.37, *p* < 0.001) and GDF-15 (*H* = 28.42, *p* < 0.001) among the three groups. Dunn–Bonferroni pairwise comparisons indicated that both VM subgroups had higher CGRP and GDF-15 levels than healthy controls (adjusted *p* < 0.0167), whereas the two VM subgroups did not differ significantly. No significant between-group differences were found for FGF-21 (*H* = 4.07, *p* = 0.130) or IL-17 (*H* = 3.07, *p* = 0.215) (Table 6). These findings indicate differences between VM cases and healthy controls but do not demonstrate that family history or genetic factors regulate serum biomarker levels.

**Table 6:** Serum biomarker levels by family-history status and healthy-control status.

Biomarker	Family-history- positive VM (*n* = 28)	Family-history- negative VM (*n* = 32)	Healthy controls (*n* = 20)	Kruskal-Wallis test	
				*H*	*P* value
CGRP, pg./mL	53.45 (21.44,78.62)^a^	51.95 (30.33,90.39)^b^	18.58 (13.23,23.66)	15.37	< 0.001
FGF-21, pg./mL	17.68 (2.80,37.02)	10.38 (3.04,33.47)	3.56 (2.64,11.82)	4.07	0.130
GDF-15, pg./mL	188.31 (110.73,322.83)^c^	205.68 (111.08,362.44)^d^	33.22 (27.11,86.96)	28.42	< 0.001
IL-17, pg./mL	134.95 (107.05,167.10)	129.88 (109.42,178.22)	122.26 (91.36,148.77)	3.07	0.215

Data are presented as median (IQR). Dunn-Bonferroni-adjusted pairwise comparisons: a, *p* = 0.002 versus healthy controls; b, P < 0.001 versus healthy controls; c and d, *P* < 0.001 versus healthy controls. Neither CGRP nor GDF-15 differed significantly between the two VM family-history groups.

### 3.5 Comparison of participants with and without available serum measurements

Among patients with VM, those with available serum measurements had higher HAMD-17 scores than those without available measurements [median (IQR): 9.0 (6.0–13.0) vs. 7.0 (4.0–10.0), *p* = 0.006]. Age, sex, BMI, hypertension, family history, DHI, H-VAS, HIT-6, HAMA-14, and MoCA-BJ scores did not differ significantly between the two VM subgroups (all *p* > 0.05).

Among healthy controls, participants with available serum measurements were younger than those without available measurements (42.85 ± 13.45 vs. 50.63 ± 12.15 years, *p* = 0.039). No statistically significant differences were observed in the other measured characteristics (all *p* > 0.05). However, several non-negligible SMDs indicated that complete comparability between participants with and without serum measurements could not be assumed (Supplementary Tables S1, S2).

### 3.6 Univariable analysis of factors associated with VM case status

In the main clinical cohort (145 VM cases and 50 healthy controls), female sex, BMI, hypertension, and positive family history differed between groups. In the biomarker subset (60 VM cases and 20 healthy controls), CGRP, FGF-21, and GDF-15 were higher in VM cases, whereas IL-17 did not differ significantly (Table 7). These analyses describe associations with case status and should not be interpreted as prospective risk factors.

**Table 7:** Univariable analysis of factors associated with VM case status.

Variable	VM group (clinical *n* = 145, biomarker *n* = 60)	Healthy controls (clinical *n* = 50, biomarker *n* = 20)	Statistic	*P* value
Age, median (IQR)	48 (37.0,53.5)	51 (37.8,58.0)	−0.215	0.830
Female, *n* (%)	126 (86.9)	28 (56.0)	21.374	< 0.01
BMI, mean ± SD	24.66 ± 3.15	23.60 ± 2.09	2.216	0.028
Smoking history, *n* (%)	20 (13.8)	2 (4.0)	3.562	0.059
Alcohol consumption history, *n* (%)	6 (4.1)	3 (6.0)	0.023	0.881
Hypertension, *n* (%)	33 (22.7)	4 (8.0)	5.268	0.022
Diabetes mellitus, *n* (%)	8 (5.5)	4 (8.0)	0.083	0.773
Coronary heart disease, *n* (%)	8 (5.5)	0 (0)	1.245	0.200
Hyperlipidemia, *n* (%)	26 (17.9)	4 (8.0)	2.817	0.093
Positive family history, *n* (%)	68 (46.8)	2 (4.0)	29.731	< 0.01
CGRP, median (IQR)	53.45 (29.09,83.66)	18.58 (13.23,23.66)	−3.917	< 0.01
FGF-21, median (IQR)	13.61 (3.04,35.47)	3.56 (2.64,11.82)	−1.991	0.047
GDF-15, median (IQR)	196.26 (110.73,332.77)	33.22 (27.11,86.96)	−5.311	< 0.01
IL-17, median (IQR)	134.22 (108.38,171.91)	122.26 (91.36,148.77)	−1.711	0.087

Clinical variables were analyzed in 145 VM cases and 50 healthy controls; serum biomarkers were analyzed in 60 VM cases and 20 healthy controls. Reported statistics are Z values for Mann–Whitney U tests, t values for independent-samples *t-*tests, and χ^2^ values for categorical comparisons.

The 145 VM cases were classified using the single family-history variable as family-history positive (*n* = 68) or family-history negative (*n* = 77). Female sex was more frequent and smoking history less frequent in the family-history-positive group. Other clinical characteristics did not differ significantly. Biomarker comparisons were based only on the smaller serum subset (28 versus 32 participants) and did not identify significant differences (Table 8). These findings support familial aggregation but do not establish a genetic mechanism.

**Table 8:** Comparison of VM cases by family-history status.

Characteristic	Family-history-positive VM (clinical *n* = 68; biomarker *n* = 28)	Family-history-negative VM(clinical *n* = 77; biomarker *n* = 32)	Statistic	*P* value
Age, median (IQR)	48.5 (38.3, 58.5)	48 (37.0, 59.0)	−0.057	0.954
Female, *n* (%)	64 (94.1)	62 (80.5)	5.864	0.015
BMI, mean ± SD	24.87 ± 2.76	24.48 ± 3.46	0.546	0.461
Smoking history, *n* (%)	5 (7.4)	15 (19.5)	4.467	0.035
Alcohol consumption history, *n* (%)	3 (4.4)	3 (3.9)	0.000	1.000
Hypertension, *n* (%)	17 (25.0)	16 (20.8)	0.366	0.545
Diabetes mellitus, *n* (%)	2 (2.9)	6 (7.8)	0.832	0.362
Coronary heart disease, *n* (%)	3 (4.4)	5 (6.5)	0.034	0.854
Hyperlipidemia, *n* (%)	13 (19.1)	13 (16.9)	0.123	0.726
CGRP, median (IQR)	53.45 (21.44, 78.62)	51.95 (30.33, 90.39)	−0.119	0.906
FGF-21, median (IQR)	17.68 (2.80, 37.02)	10.38 (3.04, 33.47)	−0.378	0.705
GDF-15, median (IQR)	188.31 (110.73, 322.83)	205.68 (111.08, 362.44)	−0.519	0.604
IL-17, median (IQR)	134.95 (107.05, 167.10)	129.88 (109.42, 178.22)	−0.445	0.657

Clinical variables were analyzed in 68 family-history-positive and 77 family-history-negative VM cases; biomarkers were analyzed in 28 and 32 cases, respectively. Reported statistics are Z values for Mann–Whitney U tests, t values for independent-samples t-tests, and χ^2^ values for categorical comparisons.

### 3.7 Exploratory multivariable logistic regression models of VM case status

Because serum biomarkers were available only in a subset of participants, two separate exploratory multivariable logistic regression models were fitted. In the clinical model comprising 145 VM cases and 50 healthy controls, age, female sex, BMI, hypertension, and positive family history were entered simultaneously. Female sex, higher BMI, and positive family history remained associated with VM case status after adjustment. Age was not statistically significant (adjusted *OR* = 1.005, 95% *CI*: 0.975–1.036, *p* = 0.766), and hypertension was also not statistically significant (adjusted *OR* = 2.988, 95% *CI*: 0.874–10.219, *p* = 0.081). In the separate biomarker model comprising 60 VM cases and 20 healthy controls, serum CGRP, FGF-21, and GDF-15 were entered simultaneously. Higher serum CGRP and GDF-15 levels remained associated with VM case status, whereas FGF-21 was not statistically significant (Table 9). Given the cross-sectional case–control design and the small biomarker subset, these findings should be interpreted as exploratory associations rather than causal risk factors or predictors of VM onset.

**Table 9:** Exploratory logistic regression models of factors associated with VM case status.

Variable	B	SE	Wald *χ^2^*	*P* value	Adjusted OR	95% *CI*	
						Lower limit	Upper limit
Model 1. Clinical model in the full cohort (145 VM cases and 50 healthy controls; n = 195)							
Age, per year	0.005	0.015	0.089	0.766	1.005	0.975	1.036
Female sex	1.514	0.434	12.196	< 0.001	4.545	1.943	10.629
BMI, per 1 kg/m^2^	0.142	0.069	4.223	0.040	1.153	1.007	1.321
Hypertension	1.095	0.627	3.045	0.081	2.988	0.874	10.219
Positive family history	2.775	0.755	13.511	< 0.001	16.031	3.651	70.384
Constant	−4.362	1.878	5.398	0.020	-	-	-
Model 2. Biomarker model in the biomarker subset (60 VM cases and 20 healthy controls; n = 80)							
CGRP, per 10 pg./mL	0.394	0.183	4.624	0.032	1.483	1.035	2.123
FGF-21, per 10 pg./mL	0.066	0.114	0.336	0.562	1.068	0.854	1.337
GDF-15, per 10 pg./mL	0.214	0.069	9.559	0.002	1.239	1.082	1.419
Constant	−2.612	0.906	8.313	0.004	-	-	-

All variables listed within each model were entered simultaneously. VM case status was coded as 1 and healthy-control status as 0. Female sex, hypertension, and positive family history were coded as 1, with male sex, absence of hypertension, and negative family history serving as the reference categories. Age and BMI were modeled as continuous variables. ORs for age represent a 1-year increase, ORs for BMI represent a 1-kg/m^2^ increase, and ORs for serum biomarkers represent a 10-pg/mL increase.

### 3.8 ROC analysis of serum biomarkers for discriminating VM cases from healthy controls

**Figure 2:**
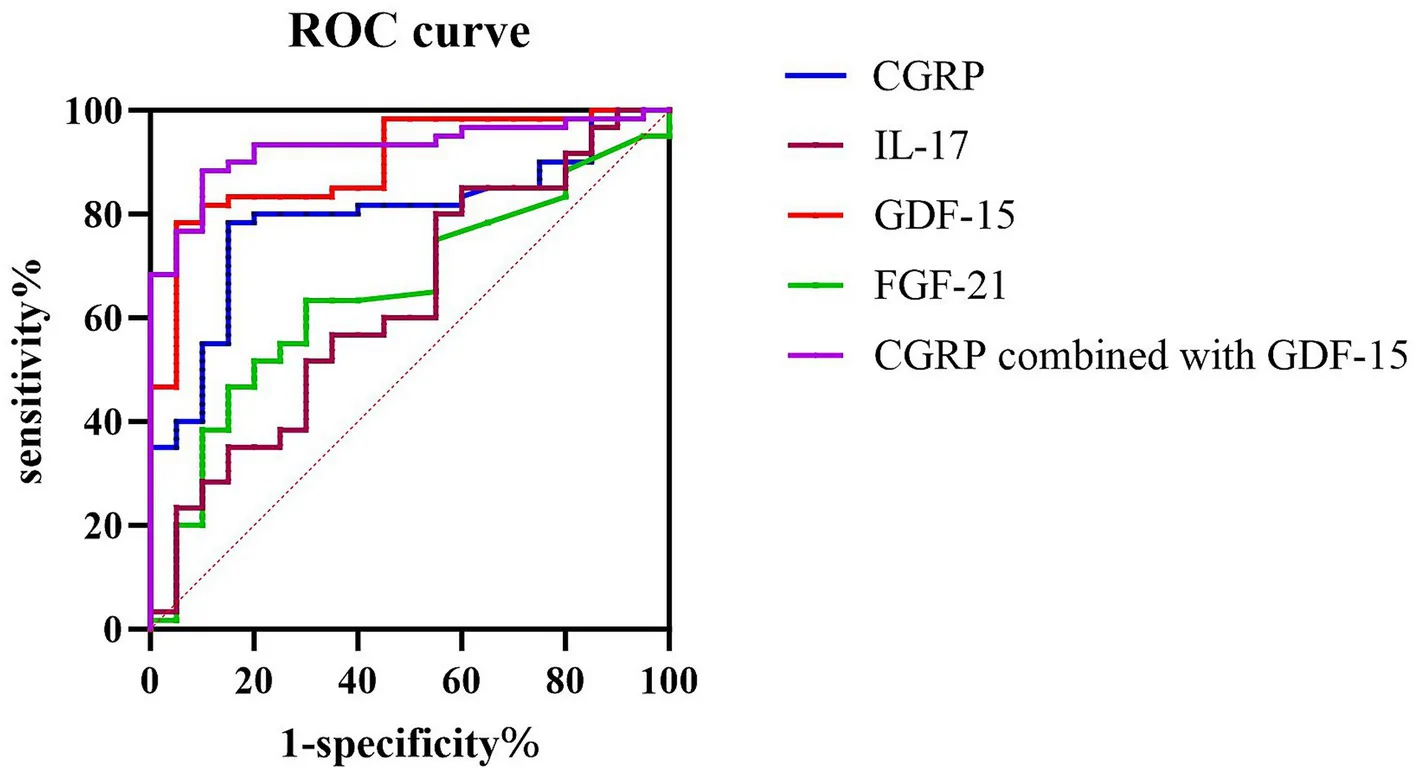
Receiver operating characteristic curves of serum biomarkers for discriminating VM cases from healthy controls. CGRP, calcitonin gene-related peptide; GDF-15, growth differentiation factor-15; FGF-21, fibroblast growth factor 21; IL-17, interleukin-17.

Within the biomarker development sample (60 VM cases and 20 healthy controls), the apparent AUC was 0.794 (95% *CI* 0.690–0.898) for CGRP and 0.898 (95% *CI* 0.826–0.971) for GDF-15. The CGRP plus GDF-15 logistic model yielded an apparent AUC of 0.927 (95% *CI* 0.869–0.985), sensitivity of 83.3%, and specificity of 90.0% at the maximum Youden index. The combined model had a higher AUC than CGRP alone (DeLong *p* = 0.006), but the difference from GDF-15 alone was not statistically significant (*p* = 0.227). Bootstrap internal validation yielded an optimism-corrected AUC of 0.918, a calibration intercept of 0.009, and a calibration slope of 0.905. FGF-21 and IL-17 showed weaker discrimination (Table 10; Figure 2). These estimates apply only to VM cases versus healthy controls in this development sample.

**Table 10:** Apparent discrimination of serum biomarkers between VM cases and healthy controls.

Test result variable	AUC	SE	Sensitivity	Specificity	Cut-off	*P* value	95% *CI*	
							Lower limit	Upper limit
CGRP	0.794	0.053	0.783	0.850	26.71	< 0.001	0.690	0.898
FGF-21	0.649	0.068	0.633	0.700	6.53	0.047	0.517	0.782
GDF-15	0.898	0.037	0.783	0.950	104.68	< 0.001	0.826	0.971
IL-17	0.628	0.072	0.850	0.400	95.86	0.087	0.486	0.770
CGRP + GDF-15	0.927	0.030	0.833	0.900	0.633*	< 0.001	0.869	0.985

AUCs are apparent estimates from the development sample (60 VM cases and 20 healthy controls). Cut-offs for individual biomarkers are expressed in pg/mL; * The cut-off value for the combined model represents the predicted-probability threshold selected by the maximum Youden index and has no measurement unit. DeLong comparisons: combined model versus CGRP, P = 0.006; versus GDF-15, p = 0.227. For the combined model, the optimism-corrected AUC was 0.918, with a calibration intercept of 0.009 and calibration slope of 0.905.

## 4 Discussion

This single-center cross-sectional case–control study characterized the clinical features of 145 patients with VM. The main clinical findings included a predominance of middle-aged women, a prolonged and recurrent disease course, frequent previous alternative diagnoses, and alternating episodes of dizziness/vertigo and headache. Emotional fluctuations, fatigue, and sleep deprivation were the most commonly reported triggering or aggravating factors. Separate exploratory multivariable analyses were conducted for clinical variables and serum biomarkers because serum measurements were available only in a subset of participants. In the full clinical cohort, female sex, higher BMI, and a positive family history remained associated with VM case status after adjustment for age and the other clinical variables. Hypertension was associated with VM case status in the univariable comparison but was not statistically significant in the adjusted clinical model. In the biomarker subset, higher serum CGRP and GDF-15 levels remained associated with VM case status, whereas FGF-21 was not significantly associated. These findings represent statistical associations and familial aggregation but do not establish causality or genetic susceptibility. In the biomarker subset, serum CGRP and GDF-15 levels were higher in patients with VM than in healthy controls and showed exploratory discrimination between VM cases and healthy individuals, both separately and in combination. However, because clinically relevant disease controls and independent validation were not included, these findings should be regarded as hypothesis-generating and should not be interpreted as evidence of differential diagnostic performance, risk prediction, or treatment guidance.

### 4.1 Demographic and clinical phenotypic characteristics of VM

Patients with VM in this study were predominantly middle-aged women, with females accounting for 86.9% of the cohort and a male-to-female ratio of 1:6.6. Participants aged 45–59 years represented 61.4% of the sample, consistent with previous reports describing a female predominance and a relatively high frequency of VM in middle-aged adults (21, 22). Sex-related biological and hormonal factors may partly contribute to this pattern. Estrogen and progesterone can influence neurotransmitter signaling, cerebrovascular regulation, and trigeminovascular pathways (23, 24). However, menstrual status, menopausal status, hormone therapy, and circulating sex hormone levels were not systematically assessed in this study. Therefore, the observed female predominance cannot be directly attributed to hormonal fluctuations and should be interpreted as a hypothesis requiring further investigation.

The mean BMI of patients with VM was 24.66 ± 3.15 kg/m^2^, and higher BMI remained associated with VM case status in the exploratory multivariable analysis (adjusted *OR* = 1.153, 95% *CI*: 1.007–1.321, *p* = 0.040). Previous studies have suggested that obesity-related metabolic abnormalities and chronic low-grade inflammation may influence migraine-related and vestibular pathways (25). Nevertheless, the cross-sectional design does not establish whether higher BMI preceded VM or resulted from associated lifestyle, metabolic, or clinical factors. Accordingly, BMI should be regarded as a clinical correlate of VM in the present study rather than a causal risk factor, and the potential effects of weight management on VM require confirmation in prospective interventional studies.

### 4.2 Clinical symptoms and reported triggering or aggravating factors

The clinical findings showed that VM commonly followed a recurrent and prolonged course. Nearly 80% of patients had a disease duration exceeding 1 year, and most reported more than five or frequent episodes of vertigo/dizziness and headache. Alternating episodes of vertigo/dizziness and headache were the most common presentation (69.7%), whereas 5.5% of patients reported isolated vertigo/dizziness. VM may coexist with PPPD, although the cross-sectional design of this study did not allow assessment of temporal progression or diagnostic transition between the two disorders (26). The observed symptom pattern may reflect interactions between vestibular and migraine-related neural pathways, but the underlying mechanisms were not directly examined in this study (27).

Nausea and/or vomiting, fatigue, and irritability were reported by more than 80% of patients. Phonophobia, photophobia, and visual aura are recognized migraine-associated features; visual aura was reported by 26 patients (17.9%) in the present cohort. Neck stiffness/discomfort and chest tightness/palpitations were also frequently reported, but these symptoms alone cannot establish cervical or autonomic dysfunction. Their overlap with other conditions may partly contribute to previous diagnoses such as cervical spondylosis, which was reported in 27.6% of patients (28).

Most patients reported at least one triggering or aggravating factor, most commonly emotional fluctuations, fatigue, and sleep deprivation. Bright light or noise and changes in environmental temperature were also frequently reported. These findings are consistent with previous reports of visual, sensory, and emotional sensitivity in VM (29, 30). However, the triggers were self-reported and may have been affected by recall bias. They should therefore be interpreted as clinical correlates rather than proven causes. Identification and individualized management of possible triggers may be useful in clinical care, although their effects on attack frequency require confirmation in prospective studies.

### 4.3 Diagnostic challenges, previous alternative diagnoses, and healthcare burden of VM

Previous diagnoses of anxiety or depression (40.0%) and cervical spondylosis (27.6%) were commonly reported by patients with VM. This may partly reflect symptom overlap with psychological disorders, cervical discomfort, BPPV, and other causes of dizziness or vertigo (31, 32). VM and Ménière’s disease may coexist or share episodic vestibular symptoms; fluctuating low-frequency sensorineural hearing loss and aural symptoms support Ménière’s disease, whereas migraine features support VM, and longitudinal audiological assessment may be required. The present study did not independently verify all previous diagnoses or directly investigate reasons for diagnostic delay; the findings should therefore be interpreted as patient-reported or record-based diagnostic patterns.

In addition, 129 of 145 patients (89.0%) reported healthcare expenditure exceeding 2,000 RMB, suggesting a considerable economic burden associated with repeated consultations, examinations, and delayed recognition. Nevertheless, because medical costs were self-reported and the observation period and cost components were not fully standardized, these findings should be interpreted cautiously. Greater awareness of VM diagnostic criteria and the appropriate use of clinical history, audiological assessment, and vestibular testing may support more accurate clinical evaluation. Cranial imaging should be performed when clinically indicated to exclude alternative neurological disorders rather than used as a routine diagnostic test for VM (33).

### 4.4 Psychological symptoms, cognitive screening findings, and functional impairment in VM

Patients with VM had significantly higher DHI, HAMA-14, and HAMD-17 scores and significantly lower MoCA-BJ scores than healthy controls. These findings indicate greater dizziness-related functional impairment, more severe anxiety and depressive symptoms, and lower cognitive screening performance in the VM group. However, HAMA-14 and HAMD-17 assess symptom severity and do not independently establish psychiatric diagnoses, while the MoCA-BJ is a screening instrument and cannot by itself confirm mild cognitive impairment.

Recurrent vertigo and dizziness may restrict daily activities and reduce quality of life, which may contribute to anxiety and depressive symptoms. Conversely, psychological distress may be associated with greater symptom perception and vestibular disability (34, 35). Nevertheless, the cross-sectional design does not allow determination of the temporal or causal relationship between VM and psychological symptoms.

Lower MoCA-BJ scores may reflect difficulties in attention, memory, or visuospatial processing, potentially related to interactions between vestibular and cognitive neural networks (36, 37). However, education, emotional status, sleep disturbance, medication use, and symptom severity may also affect cognitive screening performance. Therefore, these results should be interpreted as lower cognitive screening scores rather than evidence of confirmed cognitive impairment. Clinical management may include assessment of psychological symptoms and cognitive concerns, with further specialist evaluation when clinically indicated.

### 4.5 Clinical and biomarker factors associated with VM case status and familial aggregation

In the adjusted clinical model, female sex, higher BMI, and a positive family history were associated with VM case status. In the separate biomarker model, higher serum CGRP and GDF-15 levels were associated with VM case status, whereas FGF-21 was not significantly associated. These variables represent demographic, metabolic, familial, and serum biomarker correlates of VM. However, because of the cross-sectional case–control design, the observed associations cannot establish temporal sequence or causality and should not be interpreted as risk factors for VM onset.

A positive family history showed the strongest association with VM case status (adjusted *OR* = 16.031, 95% *CI*: 3.651–70.384, *p* < 0.001), suggesting familial aggregation of migraine- and vestibular-related symptoms. Nevertheless, family history alone does not provide direct evidence of genetic susceptibility because no genetic variants or ion-channel abnormalities were examined in this study. The higher proportion of women in the family-history-positive group may reflect sex-related differences in VM presentation or familial aggregation, but hormonal status and genetic mechanisms were not directly assessed. Further family-based and molecular genetic studies are required to clarify the relative contributions of inherited and shared environmental factors (38, 39).

Hypertension was more frequent among patients with VM than among healthy controls in the univariable comparison. Vascular dysfunction and altered trigeminovascular signaling have been proposed as possible mechanisms linking hypertension with migraine-related disorders (40). However, it was not statistically significant after adjustment for age, sex, BMI, and the combined family-history variable in the clinical multivariable model (adjusted *OR* = 2.988, 95% *CI*: 0.874–10.219, *p* = 0.081). The wide confidence interval indicates considerable uncertainty in the estimate. Residual confounding by metabolic status, medication use, and other vascular factors also cannot be excluded. Therefore, the present study does not provide sufficient evidence that hypertension is independently associated with VM case status or that blood-pressure management reduces VM attacks.

Serum CGRP and GDF-15 levels were higher in patients with VM than in healthy controls, whereas no significant differences were observed between the family-history-positive and family-history-negative VM groups. CGRP is a neuropeptide involved in trigeminovascular activation, neurogenic inflammation, and pain sensitization and may also participate in interactions between migraine and vestibular pathways (41). Nevertheless, elevated serum CGRP in this study represents an association with VM case status and does not provide direct evidence that CGRP elevation caused vestibular symptoms or that CGRP-targeted treatment would be effective in this population.

GDF-15 is a stress-response cytokine associated with metabolic stress, tissue injury, and inflammatory processes. Its elevation in patients with VM may reflect nonspecific physiological stress related to recurrent symptoms, metabolic factors, sleep disturbance, or psychological distress (42, 43). The present data do not demonstrate that GDF-15 acts directly on the vestibular nuclei or plays a causal role in VM pathogenesis. Accordingly, the findings for CGRP and GDF-15 should be considered hypothesis-generating rather than mechanistic evidence.

Overall, the adjusted analyses identified female sex, higher BMI, and a positive family history as clinical correlates of VM case status, while higher serum CGRP and GDF-15 levels were identified as serum biomarker correlates in the smaller biomarker subset. These findings do not support causal inference, genetic prediction, high-risk screening, or development of a clinically applicable predictive model.

### 4.6 Exploratory discrimination of serum biomarkers between VM cases and healthy controls

ROC analysis was performed to explore the ability of serum biomarkers to distinguish patients with VM from healthy controls in the biomarker subset. GDF-15 showed an AUC of 0.898 (95% *CI*: 0.826–0.971, *p* < 0.001), with a sensitivity of 78.3%, a specificity of 95.0%, and an optimal cut-off value of 104.68 pg./mL. CGRP showed an AUC of 0.794 (95% *CI*, 0.690–0.898, *p* < 0.001), with a sensitivity of 78.3%, a specificity of 85.0%, and an optimal cut-off value of 26.71 pg./mL. These findings suggest that CGRP and GDF-15 differed between VM cases and healthy individuals and showed exploratory discrimination within the present study sample (44–46). However, the cut-off values were derived and evaluated in the same development sample and should not be considered validated clinical thresholds.

The combination of CGRP and GDF-15 produced an apparent AUC of 0.927. DeLong testing showed that the combined model discriminated significantly better than CGRP alone (*p* = 0.006), but its AUC was not significantly higher than that of GDF-15 alone (*p* = 0.227). Bootstrap validation suggested limited optimism (optimism-corrected AUC = 0.918), although internal validation within the same small dataset does not replace external validation. Moreover, the study included only healthy controls and no patients with BPPV, Ménière’s disease, PPPD, or other clinically relevant causes of dizziness and headache. The results therefore cannot establish differential-diagnostic performance, reduction of misdiagnosis, or clinical screening utility. External validation in clinically relevant populations is required before the model can be considered for application.

In addition, serum-tested VM cases had higher HAMD-17 scores than VM cases without serum measurements, whereas serum-tested healthy controls were younger than controls without serum measurements. These selection differences may have influenced the observed biomarker comparisons and ROC estimates and further limit their generalizability.

FGF-21 showed an AUC of 0.649 (95% *CI*: 0.517–0.782, *p* = 0.047), whereas IL-17 showed an AUC of 0.628 (*p* = 0.087), indicating relatively limited discrimination between VM cases and healthy controls in this sample. FGF-21 is associated with metabolic and mitochondrial processes, while IL-17 is involved in systemic inflammatory responses (47–49). However, because no disease-control groups were included, the present study cannot determine the disease specificity of any of the measured biomarkers. These findings should therefore be regarded as exploratory and hypothesis-generating. Future studies should include larger samples, clinically relevant vestibular disease controls, prespecified models, and independent validation to determine whether CGRP, GDF-15, or their combination has additional value beyond standard clinical assessment.

### 4.7 Limitations and future directions

This study has several limitations. First, its single-center cross-sectional case–control design limits generalizability and precludes causal or temporal inference. Definite and probable VM were both included, but their numbers were unavailable; therefore, a sensitivity analysis restricted to definite VM could not be performed. Second, serum biomarkers were measured in only 60 patients with VM and 20 healthy controls. Participants with and without serum measurements differed in HAMD-17 scores among VM cases and in age among healthy controls, suggesting possible selection bias. Therefore, the biomarker subset may not be fully representative of the complete cohort. The small number of healthy controls also increased the risk of imprecision and overfitting. Third, separate clinical and biomarker regression models were fitted because biomarker data were available only in a subset. Thus, the regression and ROC findings should be considered exploratory rather than validated predictive results. Fourth, only interictal samples were collected. Medication use, fasting status, collection time, hormonal status, patent foramen ovale status, and time since the most recent episode were not systematically controlled. Analytical ranges, duplicate-measurement procedures, and intra- and inter-assay coefficients of variation were also unavailable. Self-reported triggering factors, family history, previous diagnoses, and medical costs may have been affected by recall or classification bias. The use of healthy controls without clinically relevant vestibular disease controls may have introduced spectrum bias, and residual confounding by measured and unmeasured factors cannot be excluded.

Future studies should use multicenter prospective designs, include larger samples and clinically relevant disease-control groups, record definite and probable VM separately, collect paired ictal and interictal specimens, standardize potential confounders, and perform external model validation. Such studies are required to determine whether CGRP, GDF-15, or their combination provides additional value beyond established diagnostic criteria and standard vestibular assessment.

## 5 Conclusion

In this single-center cross-sectional case–control study, VM was characterized by a predominance of middle-aged women, a recurrent and prolonged clinical course, frequent previous alternative diagnoses, and substantial functional, psychological, and economic burden. In the adjusted clinical model, female sex, higher BMI, and a positive family history were associated with VM case status, whereas hypertension was not statistically significant after adjustment. A positive family history suggested familial aggregation of vestibular symptoms but did not provide direct evidence of genetic susceptibility.

In the separate biomarker model, higher serum CGRP and GDF-15 levels were associated with VM case status, whereas FGF-21 was not significantly associated. CGRP and GDF-15 also showed exploratory discrimination between VM cases and healthy controls, both individually and in combination. Given the exploratory design and the absence of clinically relevant disease controls or external validation, the clinical utility of these findings remains uncertain. Larger multicenter prospective studies including vestibular disease controls, standardized biomarker sampling, and external model validation are needed to determine their clinical relevance.

## Data Availability

The original contributions presented in the study are included in the article/Supplementary material, further inquiries can be directed to the corresponding author.
