# Supplementary material for: Clinical characteristics and exploratory serum biomarker findings in vestibular migraine: a cross-sectional case–control study
Source: Front Neurol. 2026 Aug 26;17:1929972. doi: 10.3389/fneur.2026.1929972 (PMC13553368; doi:10.3389/fneur.2026.1929972)
Supplement: Supplementary file 1 [file Supplementary_file_1.docx]

**Supplementary Table S1.** Comparison of VM cases with and without available serum biomarker measurements

| Characteristic | With available serum biomarker measurements (n=60) | Without available serum biomarker measurements (n=85) | Statistic | *P* value | SMD |
| --- | --- | --- | --- | --- | --- |
| Age, years | 45.00 (36.00,54.25) | 49.00 (40.00,62.00) | *U*=2065.50 | 0.052 | -0.336 |
| Female sex, n (%) | 54 (90.0) | 72 (84.7) | *χ²*=0.866 | 0.352 | 0.160 |
| BMI, kg/m² | 24.90 (23.78,27.40) | 24.13 (22.49,25.97) | *U*=2962.50 | 0.098 | 0.210 |
| Hypertension, n (%) | 13 (21.7) | 20 (23.5) | *χ²*=0.069 | 0.792 | -0.045 |
| Positive family history, n (%) | 28 (46.7) | 40 (47.1) | *χ²*=0.002 | 0.963 | -0.008 |
| DHI total score | 60.00 (52.00,62.50) | 56.00 (52.00,60.00) | *U*=2896.00 | 0.163 | 0.152 |
| H-VAS score | 6.00 (6.00,7.25) | 7.00 (5.00,7.00) | *U*=2648.00 | 0.688 | 0.161 |
| HIT-6 score | 58.40 ± 6.11 | 58.82 ± 5.81 | *t*=-0.423 | 0.673 | -0.071 |
| HAMA-14 score | 10.00 (9.00,13.00) | 10.00 (7.00,12.00) | *U*=2988.50 | 0.077 | 0.280 |
| HAMD-17 score | 9.00 (6.00,13.00) | 7.00 (4.00,10.00) | *U*=3231.00 | 0.006 | 0.448 |
| MoCA-BJ score | 26.00 (25.00,27.00) | 26.00 (24.00,27.00) | *U*=2456.50 | 0.891 | 0.090 |

**Supplementary Table S2.** Comparison of healthy controls with and without available serum biomarker measurements

| Characteristic | With available serum biomarker measurements (n=20) | Without available serum biomarker measurements  (n=30) | Statistic | *P* value | SMD |
| --- | --- | --- | --- | --- | --- |
| Age, years | 42.85 ± 13.45 | 50.63 ± 12.15 | *t*=-2.126 | 0.039 | -0.614 |
| Female sex, n (%) | 14 (70.0) | 14 (46.7) | *χ²*=2.652 | 0.103 | 0.487 |
| BMI, kg/m² | 23.14 ± 2.20 | 23.92 ± 1.99 | *t*=-1.299 | 0.200 | -0.375 |
| Hypertension, n (%) | 1 (5.0) | 3 (10.0) | - | 0.641 | -0.191 |
| Positive family history, n (%) | 0 (0.0) | 2 (6.7) | - | 0.510 | -0.378 |
| DHI total score | 5.00 (4.00,6.00) | 6.00 (4.00,6.00) | *U*=271.00 | 0.554 | -0.151 |
| H-VAS score | 1.00 (0.75,2.00) | 1.00 (0.00,2.00) | *U*=312.50 | 0.804 | 0.061 |
| HIT-6 score | 38.00 (36.00,38.00) | 36.00 (36.00,38.00) | *U*=329.50 | 0.531 | 0.139 |
| HAMA-14 score | 0.50 (0.00,2.00) | 0.00 (0.00,1.75) | *U*=345.50 | 0.322 | 0.291 |
| HAMD-17 score | 1.00 (0.00,2.00) | 0.00 (0.00,1.75) | *U*=366.00 | 0.144 | 0.324 |
| MoCA-BJ score | 28.00 (27.00,29.25) | 28.00 (27.00,28.00) | *U*=375.00 | 0.128 | 0.470 |

Note: Continuous variables are presented as mean ± SD or median (IQR), as appropriate, and categorical variables as n (%). Comparisons were performed using the independent-samples t-test, Mann–Whitney U test, Pearson’s chi-square test, or Fisher’s exact test. SMD, standardized mean difference.
